# Supplementary material for: Enhanced fitness of SARS-CoV-2 variant of concern Alpha but not Beta
Source: Nature. 2021 Dec 22;602(7896):307–13. doi: 10.1038/s41586-021-04342-0 (PMC8828469; doi:10.1038/s41586-021-04342-0)
Supplement: Supplementary file 1 — Reporting Summary [file 41586_2021_4342_MOESM1_ESM.pdf]

Corresponding author(s): Charaf Benarafa
Martin Beer
Volker Thiel

Last updated by author(s): Nov 26, 2021

Reporting Summary

Nature Portfolio wishes to improve the reproducibility of the work that we publish. This form provides structure for consistency and transparency in reporting. For further information on Nature Portfolio policies, see our Editorial Policies and the Editorial Policy Checklist.

Statistics

For all statistical analyses, confirm that the following items are present in the figure legend, table legend, main text, or Methods section.

n/a	Confirmed	
☐	☒	The exact sample size (*n*) for each experimental group/condition, given as a discrete number and unit of measurement
☐	☒	A statement on whether measurements were taken from distinct samples or whether the same sample was measured repeatedly
☐	☒	The statistical test(s) used AND whether they are one- or two-sided<br>*Only common tests should be described solely by name; describe more complex techniques in the Methods section.*
☐	☒	A description of all covariates tested
☐	☒	A description of any assumptions or corrections, such as tests of normality and adjustment for multiple comparisons
☐	☒	A full description of the statistical parameters including central tendency (e.g. means) or other basic estimates (e.g. regression coefficient) AND variation (e.g. standard deviation) or associated estimates of uncertainty (e.g. confidence intervals)
☐	☒	For null hypothesis testing, the test statistic (e.g. *F*, *t*, *r*) with confidence intervals, effect sizes, degrees of freedom and *P* value noted<br>*Give P values as exact values whenever suitable.*
☒	☐	For Bayesian analysis, information on the choice of priors and Markov chain Monte Carlo settings
☒	☐	For hierarchical and complex designs, identification of the appropriate level for tests and full reporting of outcomes
☒	☐	Estimates of effect sizes (e.g. Cohen's *d*, Pearson's *r*), indicating how they were calculated

Our web collection on statistics for biologists contains articles on many of the points above.

Software and code

Policy information about availability of computer code

Data collection	ELISA: Tecan i-control 2014 1.11<br>qRT-PCR: QuantStudio™ Real-Time PCR Software (v1.7.1), or 7500 Fast System SDS Software Version 1.4<br>Viral titers: manual counting, registered in Microsoft Excel 2016 (16.0.5239.1001)<br>BLI: Octet RED96e instrument with ForteBio Data Acquisition Software (Version: 12.0.1.8)
Data analysis	relative variant quantification: Bio-Rad CFX Maestro 1.1 Version 4.1.2433.1219<br>sequence analysis: Geneious Prime ® 2019.2.3<br>figures: GraphPad Prism 8.4.2 (679) for Windows, Microsoft PowerPoint 2016 (16.0.4266.1001), Adobe Photoshop CS5 64 bit<br>NGS: Genome Sequencer Software Suite (version 2.6; Roche, https://roche.com), variant analysis tool integrated in Geneious Prime (2019.2.3)<br>digital PCR: QuantaSoft Analysis Pro software (version 1.0.596)<br>ELISA: Microsoft Excel 2016 (16.0.5188.1000)<br>Statistical analysis: GraphPad Prism version 8 or R (version 4.1), using the packages tidyverse (v1.3.1), ggpubr (v0.4.0), rstatix (v.0.7.0).<br>BLI: ForteBio Data Analysis Software (Version 12.0.1.2)

For manuscripts utilizing custom algorithms or software that are central to the research but not yet described in published literature, software must be made available to editors and reviewers. We strongly encourage code deposition in a community repository (e.g. GitHub). See the Nature Portfolio guidelines for submitting code & software for further information.

Data

Policy information about availability of data

All manuscripts must include a data availability statement. This statement should provide the following information, where applicable:

- Accession codes, unique identifiers, or web links for publicly available datasets
- A description of any restrictions on data availability
- For clinical datasets or third party data, please ensure that the statement adheres to our policy

Sequence data are available on the NCBI Sequence Read Archive (SRA) under the accession numbers PRJEB45736, and PRJNA784099, or in GenBank under the accession numbers MT108784, MZ433432, OL675863, OL689430, and OL689583 as shown in Extended Data Table 1. Source data are provided with this paper.

Field-specific reporting

Please select the one below that is the best fit for your research. If you are not sure, read the appropriate sections before making your selection.

☒ Life sciences ☐ Behavioural & social sciences ☐ Ecological, evolutionary & environmental sciences

For a reference copy of the document with all sections, see nature.com/documents/nr-reporting-summary-flat.pdf

Life sciences study design

All studies must disclose on these points even when the disclosure is negative.

Sample size	No sample size calculations were performed. Number of animals used in experiments were based on our previous comparative studies of fitness of SARS-CoV-2 variants.
Data exclusions	No data were excluded from analysis.
Replication	Binding Assays (BLI): data are representative of 3 independent experiments.<br>Airway epithelial cell (AEC) cultures in vitro: All attempts at replication were successful; experiments were performed independently on different biological donors according to best practices and as described in the Methods.<br>In vivo: Competition experiments between two VOCs were performed on groups of 6 infected animals and replicated in 4 animal models. Competition between wt-S614G and Alpha was replicated in males and females. All attempts at replication were successful. Single VOC infections were performed in groups of 8 mice for each variant.
Randomization	No randomization was required for all in vitro and in vivo competition experiments because the viral and host response parameters were measured within each cell culture insert or each animal.<br>For single infection with VOCs, hACE2-KI were randomly assigned to the respective study groups.
Blinding	Investigators were blinded during analysis of viral plaque and qRT-PCR assays of in vitro and in vivo experiments.<br>Blinding was also not relevant for in vivo competition experiments as both arms of the comparison were in single animals.

Reporting for specific materials, systems and methods

We require information from authors about some types of materials, experimental systems and methods used in many studies. Here, indicate whether each material, system or method listed is relevant to your study. If you are not sure if a list item applies to your research, read the appropriate section before selecting a response.

Materials & experimental systems

n/a	Involved in the study	
☐	☒	Antibodies
☐	☒	Eukaryotic cell lines
☒	☐	Palaeontology and archaeology
☐	☒	Animals and other organisms
☒	☐	Human research participants
☒	☐	Clinical data
☒	☐	Dual use research of concern

Methods

n/a	Involved in the study	
☒	☐	ChIP-seq
☒	☐	Flow cytometry
☒	☐	MRI-based neuroimaging

Antibodies

Antibodies used	anti-SARS nucleocapsid antibody (Novus Biologicals #NB100-56576)

Validation	relevant validation information can be accessed at https://www.novusbio.com/products/sars-nucleocapsid-protein-antibody_nb100-56576#reviews-publications

Eukaryotic cell lines

Policy information about cell lines

Cell line source(s)	Vero E6 cells (FLI): Collection of Cell Lines in Veterinary Medicine CCLV RIE 0929<br>Vero E6 cells (IVI, IFIK): cells were kindly provided by Doreen Muth, Marcel Müller, and Christian Drosten, Charité, Berlin, Germany (ATCC CRL-1586)<br>Vero-TMPRSS2 cells were kindly provided by Stefan Pöhlmann, German Primate Center - Leibniz Institute for Primate Research, Göttingen, Germany)<br>Primary human nasal cells were commercially procured from Epithelix, in Geneva, Switzerland.<br>Expi293F cells: ThermoFisher Scientific, USA.<br>A549-hACE2 cells were derived from ATCC CCL-185 and kindly provided by M. Schmolke, B. Mazel-Sanchez, and F. Abdul, Faculty of Medicine, Geneva
Authentication	in-house authentication for cell lines was not performed
Mycoplasma contamination	Cell lines were tested negative for mycoplasma contamination.
Commonly misidentified lines<br>(See ICLAC register)	None

Animals and other organisms

Policy information about studies involving animals; ARRIVE guidelines recommended for reporting animal research

Laboratory animals	Mustela putorius furo, ferrets, neutered male and female, 8 - 23 months<br>Mesocricetus auratus, Syrian hamster, male, 7-12 weeks<br>Mus musculus, mice B6.Cg-Tg(K18-ACE2)2Prlmn/J, male and female, 10-12 weeks<br>Mus musculus, mice B6.Cg-Ace2<tm1(ACE2)Dwnt>, male and female, 10-12 weeks
Wild animals	No wild animals were used
Field-collected samples	Field samples were not collected
Ethics oversight	Ferrets/hamsters: State Office of Agriculture ethics committee, Food Safety, and Fishery in Mecklenburg–Western Pomerania, Germany, registration number LVL MV TSD/7221.3-1-004/21<br>Mice: Commission for Animal Experimentation, Cantonal Veterinary Office of Bern, Switzerland, license BE-43/20

Note that full information on the approval of the study protocol must also be provided in the manuscript.
